# EHMTI-0133. Does medication overuse matter? Response to botulinum toxin type A in chronic migraine in patients with or without medication overuse

**DOI:** 10.1186/1129-2377-15-S1-G18

**Published:** 2014-09-18

**Authors:** H Zafar, M Khalil, F Ahmed

**Affiliations:** 1 Neurology Dept., Hull Royal Infirmary, Hull, UK

## Introduction

Chronic migraine (CM) affects 2% of the general population with substantial impact on quality of life. Chronic headache with medication overuse prevalence is about 65% in specialised-headache-centres based studies . Medication overuse (MO), by the International Headache Study definition, is different to Medication overuse headache (MOH) . There is no consensus as to whether pharmacological prophylaxis should be initiated once MO has been treated or both could be done simultaneously . Topiramate efficacy was found to be not affected by medication overuse, moreover the PREEMPT data also indicated a similar response between the two groups.

## Aim

To compare the efficacy of Botox in adults with MO versus CM patients without MO in real-life setting.

## Method

Adult patients with CM were offered Botox if they were not satisfactorily managed by oral preventive therapy. Botox was delivered as per the PREEMPT protocol. Headache diaries were maintained for 30 days prior to and continuously after receiving Botox (July 2010 and January 2014). Data were extracted for headache, migraine, and headache-free days . A responder was defined as one with either a 50% reduction in either headache days or migraine days or an increment in crystal clear days twice that of the baseline we compared the above mentioned variables response between CM with MO and CM without MO patients.

## Outcome

No significant differences were found between CM with MO (133 patients) and CM without MO (140 patients) in all of the above mentioned headache variants apart from medication over use days.

